# The Physiology of Man

**Published:** 1874-09

**Authors:** 


### BIBLIOGRAPHICAL.

*The Physiology of Man.* D. Appleton & Co., 549 and 551 Broadway, New York, Publishers.

The fifth and last volume of Dr. Flint's great and exhaustive work on Physiology, completes the labors of eleven years devoted to the subject, and places before the profession the fullest and latest views of the most eminent physiologists of the day on the important branches included in the title—the Special Senses, and Generation. The distinguishing feature of this work is that nothing is taken for granted. The author has thoroughly sifted and weighed the opinions of his contemporaries, and has held fast only those things which he has found to bear the test of actual experiment and personal observation.

The first chapter treats of the sense of touch, muscular sense and sensibility, the tactile corpuscles, and the venereal sense.

The second chapter takes up the olfactory nerves, their anatomy and physiology, and their relations to the sense of smelling and tasting.

Chapters III., IV., V., and VI., are devoted to the optic nerves, the anatomy of the eye, refraction, vision, the functions and mechanism of the iris, the muscles of the eyeball and eyelids, the lachrymal apparatus, etc.

Chapters VII,, VIII., and IX., deal with the auditory nerves, and the sense of hearing in all its relations, including the physics of sound, the appreciation of harmony, discord, etc. This necessarily requires a full consideration of the anatomy and physiology of the various parts of the auditory apparatus.

Chapter X. is devoted to gustation and the nerves of taste, the tongue, and its functions, facial paralysis, etc.

In Chapter XI. the author opens the subject of generation with a general view of the female sexual organs, the ovaries, Fallopian tubes, uterus, and the erectile tissue concerned.

Chapter XII. treats of the ovum and ovulation, puberty and menstruation, changes in the pregnant uterus, the corpus luteum, etc.

Chapter XIII. gives a full account of the male organs of generation, the testicles and their secretion, the glands of the urethra, and the changes in these organs from infancy to old age.

Chapter XIV. is on fecundation, the part of the male and female in the reproductive process, the entrance and destination of spermatozoids, the influence of the maternal mind on offspring, etc.

Chapter XV. treats of the segmentation of the vitellus and formation of the membranes and placents ; Chapter XVI. of the development of the embryon, and the osseous, muscular, cu, taneous; and nervous systems ; Chapter XVII. of the development of the alimentary system, the respiratory system and the face ; Chapter XVIII. of the development of the genito-urinary organs in both sexes, and of the circulatory system.

The work closes (Chapter XIX.) with an admirable view of the subject of fœtal life, a consideration of development after birth, maturity, the decay of age, death, and the resolution of the body into its original elements.

It is not too much to say of Dr. Flint's complete work that it stands alone as a comprehensive treatise on human physiology, and that its publication marks an epoch in medical literature.

